# Biogenic polymer based patches for congenital cardiac surgery: future development of implants

**DOI:** 10.3389/fcvm.2025.1540826

**Published:** 2025-02-27

**Authors:** Emma Richert, Linda Grefen, Alexandra Zorin, Julian Hubrich, Stefan Simon, Christian P. Sommerhoff, Christian Hagl, Christopher Herz, Dominik Obrist, Jürgen Hörer, Thierry Carrel, Maximilian Grab, Paul Philipp Heinisch

**Affiliations:** ^1^ Department of Congenital and Paediatric Heart Surgery, German Heart Centre Munich, Technical University, Munich, Germany; ^2^ Department of Cardiac Surgery, LMU University Hospital, LMU, Munich, Germany; ^3^ German Centre for Cardiovascular Research (DZHK), Partner Site Munich Heart Alliance, Munich, Germany; ^4^ ARTORG Centre for Biomedical Engineering Research, University of Bern, Bern, Switzerland; ^5^ Chair of Medical Materials and Implants, Technical University, Munich, Germany

**Keywords:** congenital, tissue engineering, heart disease, patches, innovation

## Abstract

**Objective:**

Despite advancements in surgical techniques, many patients born with congenital heart defects (CHD) require repeated reinterventions due to the limitations of materials used in congenital cardiac surgery (CCS). Traditional biogenic polymers, such as bovine or equine pericardium, are prone to calcification, have limited durability, and fail to adapt to the growth of infants. This study aims to address these challenges by investigating bacterial cellulose (BC) as a promising material for CCS.

**Methods:**

Variability in patch quality from previous studies was addressed by refining the production protocol taking advantage of optical density (OD) measurements. After a 72 h incubation, patches were harvested and tested mechanically with burst pressure and uniaxial strain testing. BC's biomechanical properties were further explored by modifying nutrient concentrations, creating different media groups (N10, N30, N50). Hybrid patches combining BC with electrospun polyurethane (ESP-PU) were developed using a specially designed 3D-printed flask to ensure uniform coating and integration.

**Results:**

The initial bacterial concentration significantly influenced cellulose yield and growth rate, with static cultures outperforming shaken ones. Nutrient-enriched media (N10, N30, N50) produced cellulose with greater elasticity and strength compared to standard C-Medium, with stiffness correlating to nutrient concentration. Inflation tests showed that N10 and N30 samples withstood higher pressures than N50, which, despite being stiffer, performed worse under rapid inflation. All samples, however, maintained pressures above physiological levels. Scanning electron microscopy analysis confirmed effective BC coating of PU fibres without altering BC fibre orientation or bacterial activity.

**Conclusion:**

BC patches demonstrated burst pressure resistance above 1,400 mmHg. BC's elasticity can be tailored, and in combination with ESP-PU, an innovative hybrid material can be produced, positioning BC as a promising biomaterial for future CCS implant development.

## Introduction

Congenital heart defects (CHD) remain the most prevalent congenital anomaly present in neonates, 25% of whom suffer from complex defects that require intervention within the first year after birth (1). Due to improved surgical techniques, 85% of CHD newborns are now expected to reach adulthood (2). However, many patients face significant limitations in their life expectancy and quality of life, and this is in no small part due to the materials and implants used in congenital cardiac surgery (CCS) (3). While biogenic polymers such as bovine or equine pericardium are the most widely utilized materials, they exhibit certain deficits: apart from their limited durability to withstand the constant stress and strain within the cardiovascular system over decades (3), implant calcification appears earlier and progresses faster in neonates and infants compared to older children or even adults (3, 4). Moreover, the implementation of xenografts like foreign pericardium carries the risk of infection as well as eliciting an immune response ultimately leading to rejection of the graft or implant, one of the many causes for the numerous reoperations necessary in paediatric patients (3). Though xenografts are now a highly standardised product it is precisely this characteristic that causes the lack of adaptability when it comes to the individualised correction of a complex heart defect. Besides these shortcomings, all the existing materials used in CCS still fail to address an immanent problem, especially for paediatric patients: they fail to grow and adapt to the changing demands of an infant while growing up. To date, the material addressing all these demands is yet to be developed (3, 4).

Bacterial cellulose (BC), a biogenic polymer produced by the bacterial strain Acetobacter xylinum (A. xylinum), shows great promise in potentially overcoming many existing challenges in the field of CCSbased on recent studies. Its ultra-fine nanofibril structure, combined with a high water-binding capacity, renders BC comparable to collagen (5). In both, *in-vitro* and *in-vivo* evaluations in animals, BC-based implants have been shown to provoke neither acute nor chronic inflammation (6, 7). Additionally, BC exhibits excellent mechanical properties, which have facilitated its successful application in large animal models for coronary bypass grafting and ventricular septal defect (VSD) Closure (5, 6, 8).

Our objective for this study was to harness BC specifically for use in CCS. We investigated the potential for improving patch quality by further refining the production process, with particular attention to inter-batch reproducibility and material characterisation. Considering the unique aspects of bacterial metabolism, we explored possibilities for tailoring mechanical properties according to different patient-specific applications within CCS focusing on composition of the used bacterial medium and physical influences. Furthermore, we explored the possibility of combining BC with electrospun polyurethane (ESP-PU) to achieve synergistic effects in terms of biomechanics and enabling the generation of more complex geometries.

## Methods

### Data availability statement

The data that support the findings of this study are available from the corresponding author upon reasonable request.

### Bacterial strain

For all experiments *Komagateibacter sucrofermentans* (DSM Lot No. 15973-0821-001, Leibniz Institute DSMZ—German Collection of Microorganisms and Cell Cultures GmbH, Braunschweig, Germany) was used. The freeze-dried colonies were set up according to the accompanying protocol provided by DSMZ using ATCC Medium 459. The bacterial solution was then incubated for 48 h at 30°C to multiply. Subsequently, the medium was changed, and the culture left to incubate for another 48 h. The resulting cellulose was then used for experiments or kept in continuous culture.

### Production of bacterial cellulose patches

**Figure 1:**
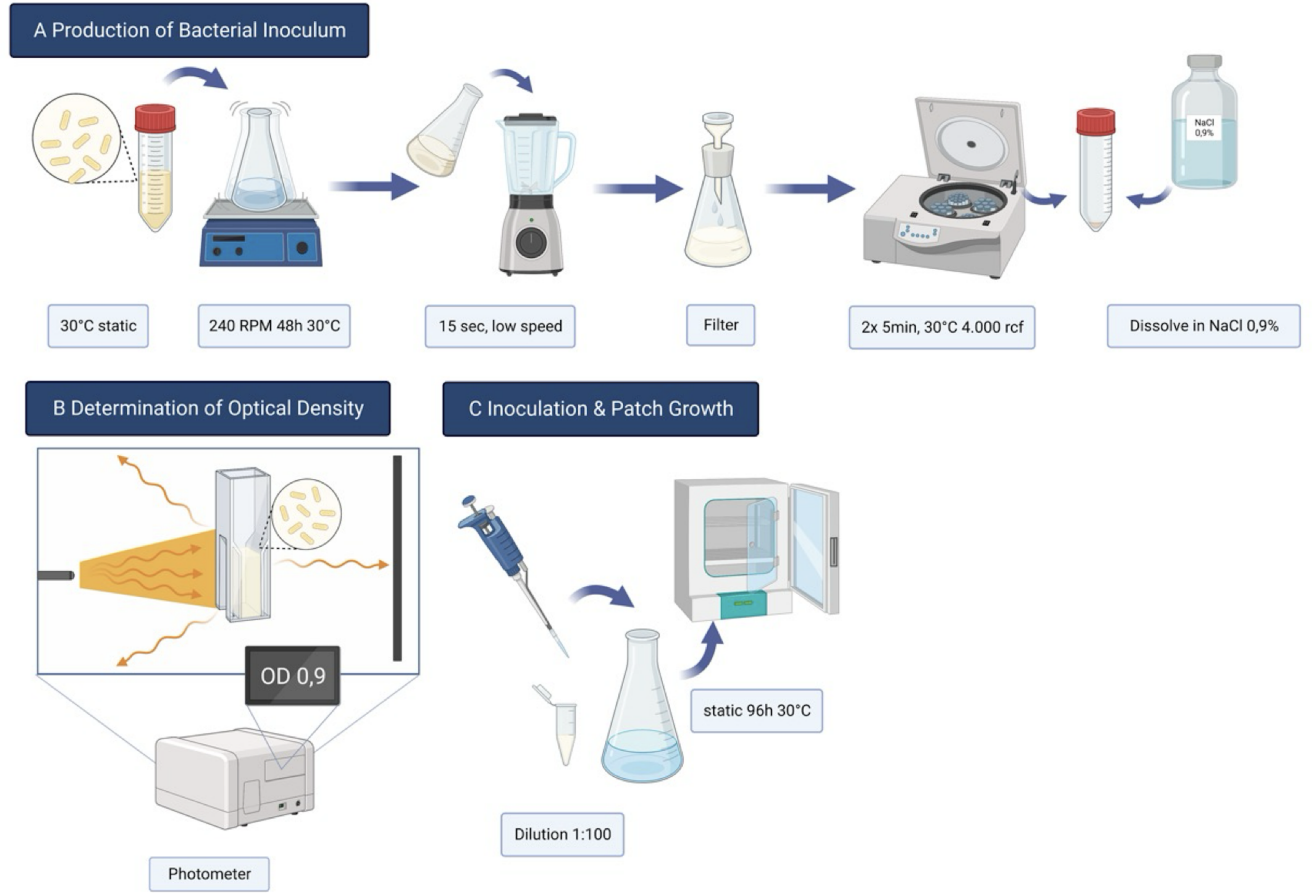
**(A)** Fabrication of bacterial inoculum for experiments. **(B)** optical density is measured by light absorbance by the bacteria in the solution. **(C)** inoculation of the Nourishing Medium and Incubation during 96 h growth.

A 1 × 1 cm piece of cellulose from the continuous culture was picked, dissolved in 200 ml of C-Medium in a 500 ml Erlenmeyer flask and left to incubate on a shaker at 240 RPM and 30°C for 48 h (see Figure 1A). Until then, the bacteria had sufficiently multiplied and the solution, now termed Inoculum, was mixed up and shredded for 15 s in a stand mixer on low speed. Next, the Inoculum was filtered through 2 layers of sterile medical gauze, centrifuged, and washed with sterile NaCl 0,9% solution twice for 5 min at 4,000 rpm using a centrifuge (Heraeus® Labofuge® 400R, Thermo Fisher Scientific Inc., Waltham, MA, USA). With the previously reported protocol (9), significant variations in cellulose patch quality were observed, evident as visible unevenness and inconsistent experimental performance. To address this, a standardisation method was implemented: optical density (OD) measurements were used to provide information about the concentration of particles in a defined volume of liquid.

OD is a logarithmic measurement of the percent transmission (%T) and can be expressed as the term A = log10(100/%T). However, OD measurements assess turbidity and are therefore suitable only for microbial colonies of low densities; they become less reliable as the OD value increases >1.0, i.e., as the proportion of transmitted rays decreases (10). Consequently, OD values were measured at 600 nm wavelength in a 1 cm cuvette using a Photometer (BioPhotometer Eppendorf AG, Hamburg, Germany) and adjusted to a value of 0,9 ± 0,25 (see Figure 1B). A series of 500 ml Erlenmeyer flasks were inoculated using a dilution Inoculate: C-Medium of 1:100 and an incubation temperature of 30°C under static conditions. After 72 h, the patches where harvested and the bacteria and media residues removed according to the postproduction protocol reported earlier (9).

Additional media were tested in comparison to the standard C-Medium. These differed in their share of nutritious ATCC Medium 459: either 10%, 30% or 50% nutrient solution was added and the results of the groups—now termed N10, N30, and N50—were compared.

### Mechanical testing

**Figure 2:**
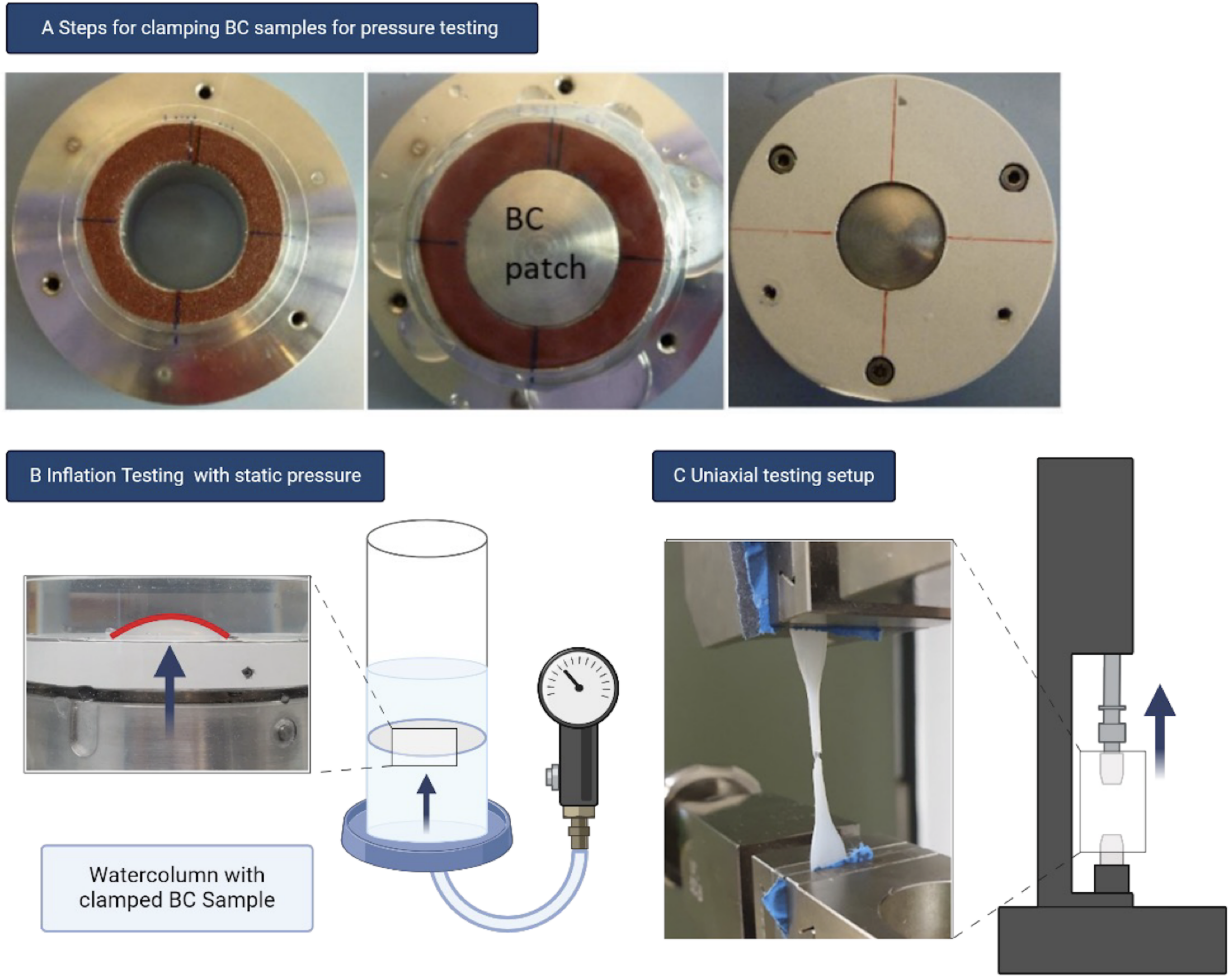
**(A)** BC sample is clamped between sandpaper rings before fixed in cylinder. **(B)** Pressure by the watercolumn is applied from below and recorded until rupture. **(C)** Samples are clamped between sandpaper before upper part is pulled upwards.

For burst pressure resistance testing BC sample was cut and clamped between two layers of sandpaper (Figure 2A) before being positioned in a cylinder and fixed with screws at a tightening torque of 1,5 Nm (C100 Medium only) and 2,5 Nm (N10, N30 and N50 group) respectively to prevent the cellulose patches from shifting. If no air bubbles occurred, the system was assumed to be sealed and the patch was inflated by water pressure from below until rupture (Figure 2B). This was recorded using a pressure sensor (WIKA A-10 PE81-60 1,6 bar, WIKA Alexander Wiegand SE & Co. KG, Klingenberg, Germany) and Coolterm software. If the sample withstood the maximum pressure of 1,400 mmHg for 2 min, it was classified as “*no rupture during experiment*”.

For uniaxial tensile testing BC samples were cut in a dogbone shape (Mould DIN 53504-S2A) using a manual punch (ZCP 020, Zwick GmbH& Co. KH, Ulm, Germany), and subsequently fixed between sandpaper in the vise grips of a tensile testing machine (zwickiLine, 2.5 kN, Zwick GmbH & Co. KG) to minimize slipping. Testing was recorded using the corresponding software (testXpert V12.3, Zwick GmbH & Co. KG) and carried out until mechanical failure. The point of failure was visually inspected and deemed valid if it occurred in the central third of the samples (Figure 2C).

### Hybrid cellulose patch development

To evaluate the compatibility of BC with other materials, it was combined with ESP-PU patches, a biocompatible material capable of mimicking the characteristics of native extracellular matrix (13). Electrospun scaffolds were produced as previously described (14): for the spinning solution a 1:1 mixture of tetrahydrofuran (THF, Tetrahydrofuran ≥99,9% Sigma-Aldrich Chemie GmbH, Taufkirchen, Germany) and dimethylformamide (DMF, N,N-Dimethylformamide ≥99.8% Sigma-Aldrich Chemie GmbH) with 15% w/v polyurethane (Pellethane 2363 80AE, Velox GmbH Hamburg, Germany) was dissolved overnight on a stirrer. This solution was loaded into a syringe pump fitted with a large-gauge needle (13G) and charged with +12.0-18.0 kV at a flow rate of 3 ml/h, with a working distance of 25 cm. Spinning was conducted at 20%–40% relative humidity and a temperature between 21 and 24°C. After the static collector achieved the desired fibre thickness within a runtime of 90–120 min, the aluminium foil was removed from the collector and placed under a fume hood for 24 h to evaporate. To prevent contamination of the bacterial culture, the pre-fabricated PU patches were soaked in 80% ethanol for 2 h, dried at 50°C for at least 6 h, and subsequently used for experiments.

**Figure 3:**
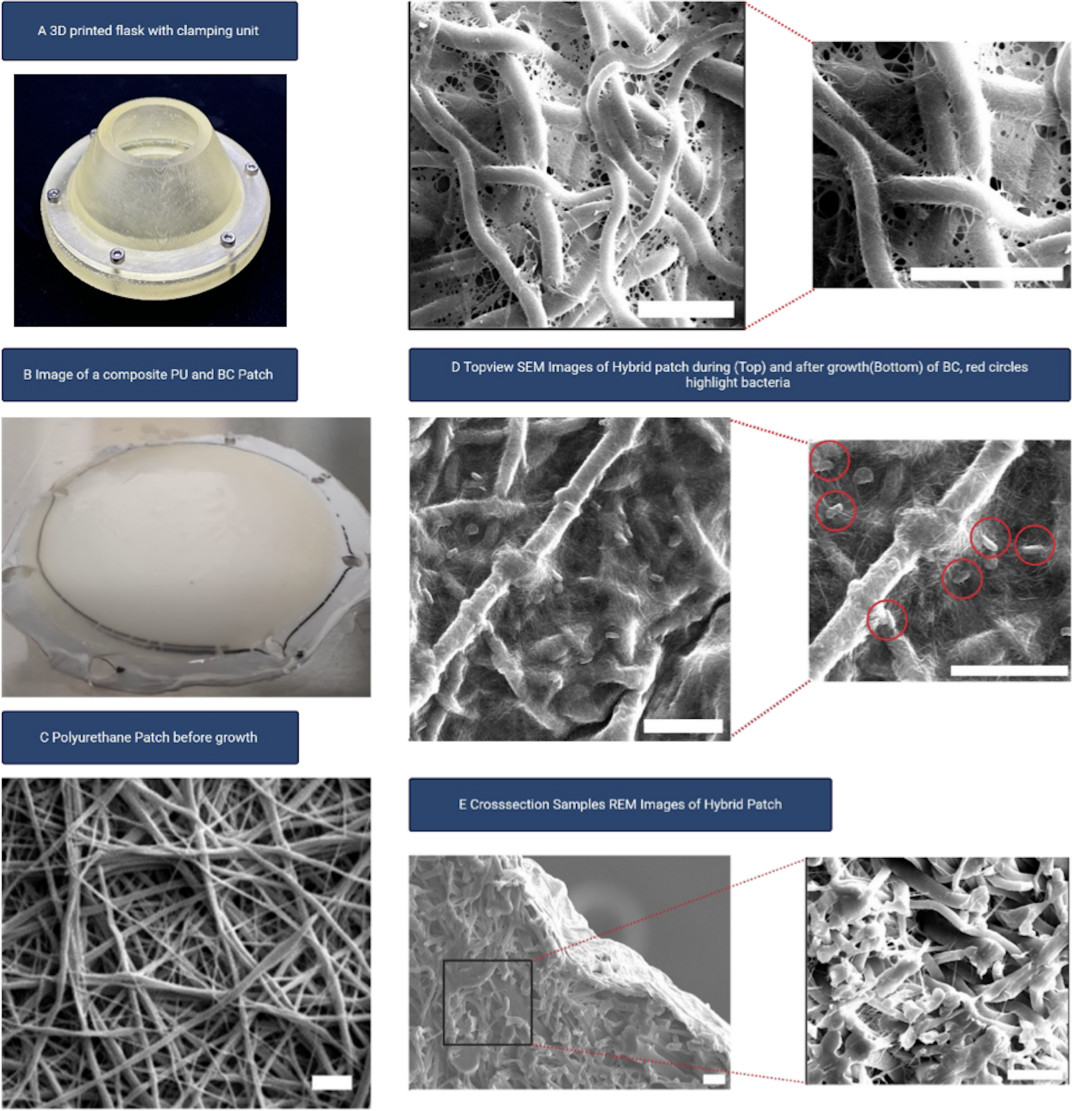
**(A)** Image of 3D printed flask including clamping unit. **(B)** Photo of a newly manufactured composite ESP-PU BC patch. **(C)** SEM Image of ESP-PU without BC, scale bar 10 µm. **(D)** Top SEM images of hybrid patch during growth, scale bar 10 µm. **(D)** Bottom SEM images of hybrid patch after growth period, red circles highlight bacteria, scale bar 10 µm. **(E)** cross-section samples SEM Images of hybrid patch, scale bar 10 µm.

To facilitate the combination of ESP-PU with BC patches, we developed a specialised manufacturing flask. In previous experiments, adding the PU patch on the surface did not yield sufficient results; it needed to be submerged to achieve adequate coating. Therefore, we 3D-printed a specially designed flask with a clamping mechanism to keep the PU patch below the surface (Figure 3A). This design ensured a uniform coating and optimal integration of the PU patch. Furthermore, the surface-to-volume ratio was set to 0,05 mm^−1^ as previous studies had demonstrated that this ratio enhances production rates and facilitates faster bacterial growth (15). Additionally, the flask was designed with a conical shape to minimize friction during the production process, commonly known as the wall effect (16).

The 3D cultivation flask was designed using CAD software (Inventor Professional Pro 2022, Autodesk Inc., San Francisco, California, USA). To manufacture the parts, a commercial SLA printer (Form 3BL, Formlabs Inc., Somerville, MA, USA) was used with a rigid, biomedical resin [Surgical Guide v1 (FLSGAM01), Formlabs Inc.]. Post-processing included removal of the support structures and washing of the parts in isopropanol (2-Propanol, Rotipuran® ≥ 99,8%, Carl Roth GmbH + Co. KG, Karlsruhe, Germany) for 20 min. The parts were dried at room temperature for 12 h. Subsequently, the models were cured for 30 min at 60°C using a commercial curing chamber (Form Cure, Formlabs Inc.). Subsequently, the 3D flasks were sterilized at 134°C for 4 min and stored in a sterile environment until usage.

After drying the hybrid patch samples at 50°C for 24 h and gold coating with a sputter (Bal-Tec, SCD 050, Balzers, Liechtenstein) image acquisition was performed using a scanning electron microscope (Zeiss Evo LS10, Carl Zeiss, Germany) to assess surface and fibre orientation.

### Data analysis & statistics

The stress strain curves obtained from the uniaxial mechanical tractor unit were exported via Excel in form of spreadsheets, which were then, along with all other experimental data records cleaned and visualised using R code. The direct group comparison was performed using *t*-test and a *p*-value < 0.05 was considered statistically significant.

## Results

### Implementation of a reproducible standard in cellulose patch quality

**Figure 4:**
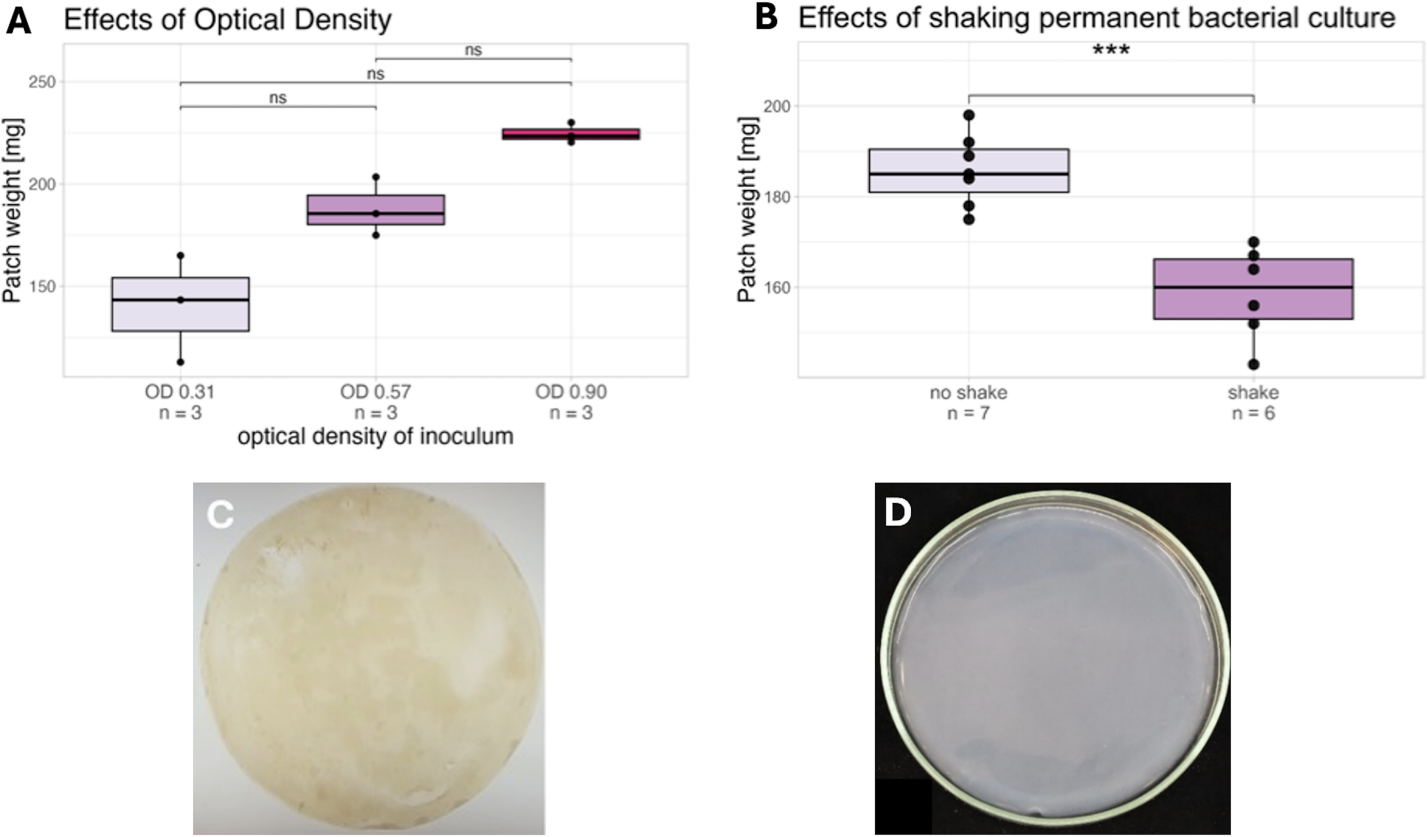
**(A)** Influence of different optical densities on the resulting cellulose yield (dried weight). **(B)** Influence of continuous shaking of the permanent bacterial culture on BC yield (dried weight). **(C)** Cellulose patch grown using former protocol (left) vs **(D)** amended inoculum Production (right).

The resulting cellulose patches were harvested after 48 h, dried at 50°C, and the weights compared (Figure 4A). The initial concentration not only influenced the amount of cellulose produced but also the growth rate, with Group OD = 0.9 being the only samples with a continuously closed surface after 48 h. Additionally, we explored the hypothesis, that stirring the permanent bacterial culture reduces its capacity to produce sufficient cellulose (17). We therefore compared a batch of BC of flasks (*n* = 7) inoculated with a static permanent bacterial culture with cellulose patches (*n* = 6) derived from shaken permanent bacterial culture (Figure 4B). Although they were subjected to the same growth conditions, the previously shaken bacteria produced significantly less cellulose as the static culture and yielded distinctly less dried BC than the comparison group. Further adjustments to the production protocol, such as mixing, filtering, and washing (see Figure 1A) enabled the removal of metabolic products of the growing bacteria, which hinder production rates (18, 19). These adjustments also achieved a more even inoculation of the flasks, resulting in smoother surfaces of the cellulose specimens (see Figures 4C,D).

### Mechanical testing of bacterial cellulose patches

**Figure 5:**
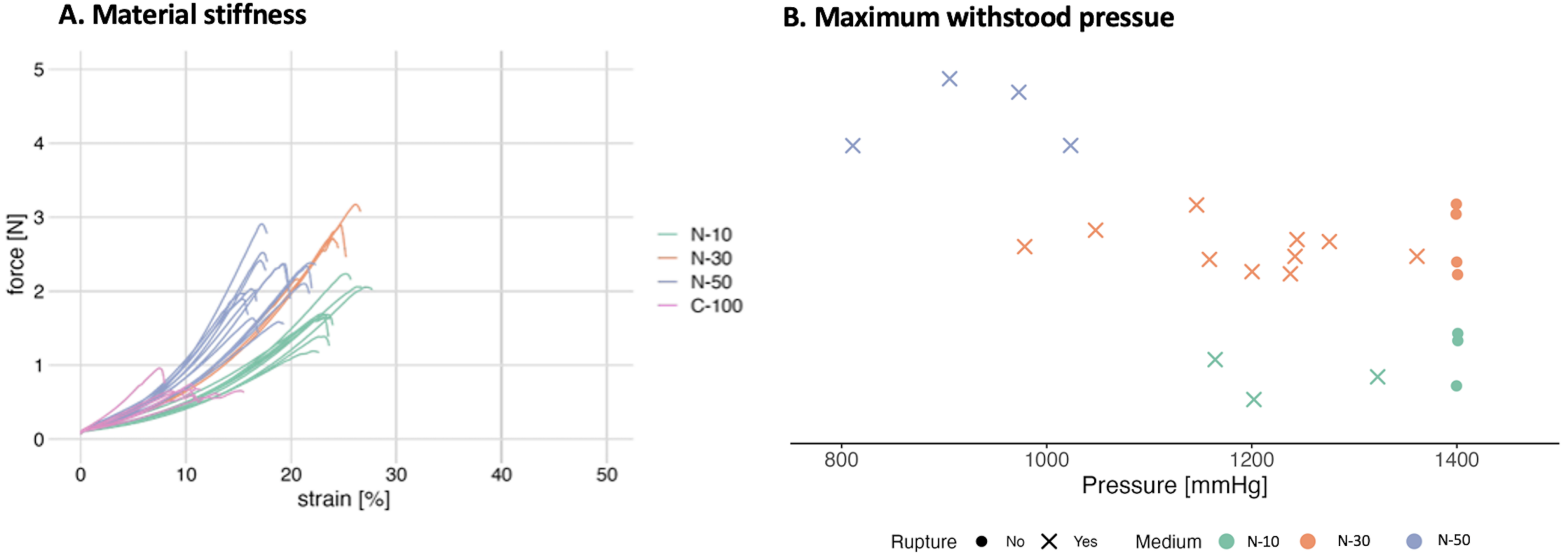
**(A)** Depending on the nutrient concentration of the different Media (N10 vs N30 vs N50) samples were stiffer or more elastic. **(B)** Maximum pressures withstood grouped by different Media (N10 vs N30 vs N50) + Median.

All samples from groups N10, N30 and N50 outperformed the previous standard C-Medium when subjected to strain-stress testing (Figure 5A). Cellulose from C-Medium already ruptured at strains of 10% whereas N10, N30 and N50 withstood 2–3 times higher forces and were evidently more elastic, with strains up to 25%. On the other hand, the stiffness of the cellulose could be determined via the nutrient concentration: N50 samples exhibited distinctly higher rigidity than N10 samples. The individual groups showed only minor deviations within, and this effect was further reproducible between different cellulose batches.

The impact of varying media concentrations was also apparent in the inflation testing setup. The N10 and N30 groups exhibited an average maximum resistance pressure of 1,200 mmHg, whereas the N50 samples only sustained pressures ranging from 800 to 1,000 mmHg (see Figure 5B). The significantly poorer performance of the N50 samples can be attributed to their increased stiffness, as observed in the previous experiment. This discrepancy is likely due to the experimental setup, where the rapid inflation may have been too abrupt for the stiffer material, leading to a distortion of the results. Nonetheless, the achieved pressures are well above physiological levels. Furthermore, 40% of the patches from the N30 group and 29% of the patches from the N10 group were able to withstand a pressure of 1,400 mmHg for at least 2 min without rupturing (Figure 5B).

### Hybrid cellulose patch development

With this setup, the production process was significantly reduced from 72 h to 48 h. Figure 3B shows an image of a newly obtained hybrid patch. Using scanning electron microscopy (SEM), it was demonstrated that the individual fibres of the PU patches were gradually encased by the bacteria during the growth process. Initially, gaps were present (Figure 3D Top), but these were fully covered after 48 h and extended growth periods only resulted in a thicker BC coating (Figure 3D Bottom). This suggests that neither fibre thickness nor the PU itself presents an adhesion problem for the bacteria; rather, the fibres served as a template for bacterial growth. Compared to pure BC patches, the fibre orientation remained random, indicating that the PU fibres did not negatively affect this aspect. Even after 48 h, visually metabolically active bacteria were still detectable in the composite patch (Figure 3D Bottom inset), suggesting that the pre-sterilization and evaporation steps were effective in removing toxic residual substances from electrospinning. Cross-sectional SEM images further revealed that the bacteria did not occupy every gap between the PU fibres. Instead, they traversed the scaffold via the most direct routes to coat the opposite surface sufficiently (Figure 3E).

## Discussion

### Shortcomings of conventional biogenic polymers and the emerging potential of bacterial cellulose

Currently, both biogenic and synthetic materials are used in the reconstruction of congenital heart defects (CHD). However, synthetic materials like DACRON and Gore-Tex® have largely been supplanted by biogenic polymers, such as autologous, equine, or bovine pericardium, due to the higher incidence of thromboembolic events and short-term infections associated with the former (3). Despite this shift, the heightened immune response in infants and altered calcium metabolism have led to acute graft rejection and premature calcification, issues less prevalent in adults (4). Thus, there is still a significant need for new patch materials to be developed.

BC is a hydrogel produced extracellularly by *Acetobacter xylinum* in liquid media near the surface, where its production relies on adequate oxygen supply (11). Beyond its impressive mechanical properties and high viscoelasticity, BC's fibril network closely resembles collagen and has demonstrated excellent biocompatibility with minimal local inflammatory response (6, 7). Lang et al. demonstrated that BC is suitable for suturing and closing ventricular septal defects using a transcatheter approach in a pig model. Histopathological analysis showed minimal inflammatory response, along with neoendothelialisation and early cell organisation (8). In another study by Fusco et al., BC was evaluated for use in small-diameter vascular grafts for coronary artery bypass grafting in a pig model, with promising results. After one month, coronary angiograms revealed that all eight grafts maintained patency without signs of dissection or rupture. The explanted grafts displayed a three-layered wall structure similar to native vessels, with various host cells, including endothelial and smooth muscle cells, indicating successful engraftment (6).

However, a significant gap remains in the available data regarding the comprehensive assessment of the material's elasticity under diverse physiological and mechanical conditions. Additionally, a deeper understanding of the bacterial metabolism involved in the production of BC is needed to optimise and harness its properties for advanced engineering applications. Further research is essential to elucidate these aspects, which are critical for fully exploiting the potential of BC in a reproduceable way in cardiac tissue engineering.

The objective of the present study was to adapt BC for use in CCS by enhancing patch quality through improvements in the production process, with a focus on ensuring consistent results across different batches and thoroughly characterising the material. We explored the unique metabolic characteristics of the bacteria to fine-tune the mechanical properties of BC, particularly by adjusting the bacterial growth medium and physical conditions during the growth period. Additionally, we investigated the potential of combining BC directly with electrospun polyurethane (ESP-PU) by letting the bacteria use the pre-fabricated ESP-PU patches as scaffolds.

### Implementation of a standardised production protocol for bacterial cellulose patches ensured consistent mechanical testing results

While previous studies were able to demonstrate the influence of nutrient concentration and media composition on yield, these adjustments where primarily economically motivated and the quality of the resulting BC often not mechanically evaluated (11, 12). The other key component researchers focus on is oxygen supply as it is widely agreed that it plays a major component in bacterial metabolism (20). Studies have shown that oxygen is not the limiting factor during extended cultivation periods. In fact, excessive oxygen supply, as seen in agitated and highly aerated cultures compared to static ones, may reduce bacterial productivity by promoting the growth of cellulose-negative clones (16, 17). The findings from this study comply with Krystynowicz et al. showing an increase in dried weight as well as a smoother surface and material (Figure 4B). Additionally, to our knowledge, we are the first group to report a dose-dependent variation in elasticity: the proportion of nutritious ATCC Medium 459 directly influenced the stiffness of the resulting samples and this effect was reproducible between batches (Figure 5A). Bodin et al. previously demonstrated that oxygen supply influences burst pressure resistance, with a reported maximum of 880 mmHg (21). In contrast, our nutrient-based approach surpassed this limit, achieving pressures exceeding 1,400 mmHg. Notably, while excessive oxygen supply in their study reduced elongation at break from 30% to 10%–20% (21), our method increased the maximum strain at peak force from 10% to 20%–30%, depending on the sample group (Figure 5A), suggesting a different underlying mechanism for this effect. Future evaluations will reveal if this effect can be attributed to a single component in the medium or if it results from a more complex mechanism. Regardless, the implications of this finding are significant for the clinical applicability of BC, as it suggests the potential to tailor its properties for various applications in congenital cardiac surgery (CCS).

### Innovative approaches: combining BC with electrospun polyurethane

BC alone may lack the biomechanical properties needed for all congenital cardiac surgery applications. To overcome this limitation, we explored combining BC with other materials to harness synergistic effects, reducing the weaknesses of each individual material. Electrospinning (ESP), a widely adopted technique in tissue engineering, was used to fabricate scaffolds, allowing the integration of diverse materials while customising fibre diameters, orientations, and porosities for enhanced performance.

Moreover, numerous postprocessing methods can further enhance tissue remodelling, improve hemocompatibility, and regulate the biodegradation of electrospun scaffolds (13, 14, 22). Additionally, ESP has been effectively employed in a core/shell configuration to produce polymers with electroactive properties, which promote normal cardiomyocyte function (13, 22). Conversely, while PU alone can be hydrophobic, it is not fully impermeable (13). Therefore, in high-pressure applications such as ascending aortic vascular prostheses, a coating might be advantageous. It has been reported that co-spinning PU and BC is feasible, but this method is time-intensive and requires a complex postproduction process (23). In contrast, our approach leverages the natural growth behaviour of the bacterial strain, and with the newly designed 3D-printed flask, the entire production process was completed in just 48 h—the same amount of time required for BC-only patches. Our setup demonstrated that a thorough understanding of bacterial growth behaviour can naturally and in a low-cost setup yield a fully coated product, with bacteria utilising the pre-existing PU fibres as growth paths. Notably, this process did not adversely affect the metabolically active bacteria, as the BC fibre orientation remained unaffected (Figure 3D). As this was primarily a feasibility study, mechanical data have not yet been collected. However, the promising preliminary results justify further investigation into postproduction methods for removing residual bacteria after the growth process. The high temperatures used in our current method risk compromising the PU and may not fully penetrate the hybrid material. Therefore, we are now exploring the effectiveness of prolonged lye treatment at lower temperatures as a potential alternative.

### Limitations and outlook

As with any experimental design, the reported sample sizes per group are limited, and variability in patch quality remains a challenge. One potential reason for this variability could be that optical density (OD) measurements, which may be affected by the size and shape of the bacteria, cannot differentiate between living and dead cells. Additionally, the centrifugation step in the production protocol presents a challenge: excessive centrifugal force may kill more bacteria than it effectively removes debris and waste medium particles.

Optimising centrifugation conditions is essential and must be customised for each specific centrifuge. However, this approach may not be ideal, and further research is needed to refine these steps. Our study only conducted static pressure testing, achieving levels well above physiological norms (see Figure 5B), but long-term durability remains untested. To address this gap, future studies should incorporate high-frequency cycling tests to evaluate material performance over extended periods.

By implementing a 3D-printed manufacturing flask, we were able to expedite the research process through rapid prototyping and the customisation of all components tailored to our experimental needs. Recognising the critical importance of maintaining adequate nutrient supply during the growth period, we propose integrating a pumping mechanism to ensure a consistent glucose supply and efficient waste removal. The flexibility of 3D printing makes it straightforward to incorporate such enhancements. Additionally, it also offers the scalability needed to increase sample sizes, thereby enhancing the reliability of future results. While the initial aim of our study was to manufacture patches for CCS, the newly reported nutrient dose-dependent variation in elasticity (Figure 5A) allows a variety of clinical applicabilities such as aortic grafts or heart valves. 3D printed manufacturing flasks will simplify the prototyping for these next steps.

## Conclusion

Bacterial cellulose (BC) holds great potential for congenital cardiac surgery (CCS) due to its customisable properties. By using a standardised production protocol, BC patches exhibited consistent mechanical strength, with burst pressures exceeding 1,400 mmHg. Its elasticity can be tailored by adjusting nutrient levels, enabling application-specific adaptations. The integration of electrospun polyurethane (ESP-PU) with BC further enhances its properties, making it more versatile. Additionally, 3D printing allows for direct customisation to individual patient anatomy, emphasising BC's clinical applicability and positioning it as a promising material for CCS implants.

## Data Availability

The raw data supporting the conclusions of this article will be made available by the authors, without undue reservation.
